# Ethanol extracts from the branch of *Taxillus yadoriki* parasitic to *Neolitsea sericea* induces cyclin D1 proteasomal degradation through cyclin D1 nuclear export

**DOI:** 10.1186/s12906-018-2258-x

**Published:** 2018-06-20

**Authors:** Su Bin Park, Gwang Hun Park, Ha Na Kim, Hun Min Song, Ho-Jun Son, Ji Ae Park, Hyun-Seok Kim, Jin Boo Jeong

**Affiliations:** 1 Department of Medicinal Plant Resources, Andong National University, Andong, 36729 Republic of Korea; 2 Forest Medicinal Resources Research Center, National Institute of Forest Science, Yongju, 36040 Republic of Korea; 3 Baekdudaegan National Arboretum, Bonghwa, 36209 Republic of Korea; 4 Department of Food Science & Biotechnology, Kyonggi University, Suwon, 16227 Republic of Korea; 5 Agricultural Science and Technology Research Institute, Andong National University, Andong, 36729 Republic of Korea

**Keywords:** Anticancer, Cell growth arrest, Cyclin D1, Mistletoe, *Taxillus yadoriki*

## Abstract

**Background:**

Although the inhibitory effect of mistletoe on cancer cell growth has been reported, the underlying mechanisms to explain its anti-proliferative activity are not fully studied. Thus, we elucidated the potential molecular mechanism of the branch from *Taxillus yadoriki* (TY) parasitic to *Neolitsea sericea* (NS) (TY-NS-B) for the anti-proliferative effect.

**Methods:**

Anti-cell proliferative effect was evaluated by MTT assay. The change of cyclin D1 protein or mRNA level was evaluated by Western blot and RT-RCR, respectively.

**Results:**

In comparison of anti-proliferative effect of TY from the host trees such as *Cryptomeria japonica* (CJ), *Neolitsea sericea* (NS), *Prunus serrulata* (PS), *Cinnamomum camphora* (CC) *and Quercus acutissima* (QA), TY-NS showed higher anti-cell proliferative effect than TY-CJ, TY-PS, TY-CC or TY-QA. In addition, the anti-proliferative effect of branch from TY from all host trees was better than leaves. Thus, we selected the branch from *Taxillus yadoriki* parasitic to *Neolitsea sericea* (TY-NS-B) for the further study. TY-NS-B inhibited the cell proliferation in the various cancer cells and downregulated cyclin D1 protein level. MG132 treatment attenuated cyclin D1 downregulation of cyclin D1 protein level by TY-NS-B. In addition, TY-NS-B increased threonine-286 (T286) phosphorylation of cyclin D1, and the mutation of T286 to alanine (T286A) blocked cyclin D1 proteasomal degradation by TY-NS-B. But the upstream factors related to cyclin D1 degradation such as ERK1/2, p38, JNK, GSK3β, PI3K, IκK or ROS did not affect cyclin D1 degradation by TY-NS-B. However, LMB treatment was observed to inhibit cyclin D1 degradation by TY-NS-B, and T286A blocked cyclin D1 degradation through suppressing cyclin D1 redistribution from nucleus to cytoplasm by TY-NS-B. In addition, TY-NS-B activated CRM1 expression.

**Conclusions:**

Our results suggest that TY-NS-B may suppress cell proliferation by downregulating cyclin D1 protein level through proteasomal degradation via T286 phosphorylation-dependent cyclin D1 nuclear export. These findings will provide the evidence that TY-NS-B has potential to be a candidate for the development of chemoprevention or therapeutic agents for human cancer.

## Background

Mistletoe as a parasitic plant which grows attached to the stems of various host trees, has been used as the traditional medicine for the treatment of several health problems including hypertension, elevated blood lipids, immune modulation, diabetes mellitus, arthritis and rheumatism in Europe and Asia [1]. In Korea, there are five taxa of four genera in two families of mistletoe: *Viscum coloratum* (Komarov) Nakai f. *coloratum*, *Viscum coloratum* (Komarov) Nakai f. *rubroaurantiacum* (Makino) Kitagawa and *Korthalsella japonica* (Thunb.) Engl. in the Santalaceae family, along with *Loranthus tanakae* Franch. et Sav. and *Taxillus yadoriki* (Sieb. ex Maxim.) Danser in the Loranthaceae family [1, 2].

Mistletoe has been reported to have a variety of the pharmacological activities such as anti-cancer, anti-inflammation, anti-HIV and immunomodulatory activities [3–6]. Among these pharmacological properties of mistletoe, mistletoe’s main application has been known for treatment of cancer therapy [7] and considered as a potent complementary and alternative medicine for various human cancer [8–10].

Regarding to the accumulating evidence for the anti-cancer activity, mistletoe exerts anti-cancer property through various mechanisms such as the cell growth arrest [11], induction of apoptosis [12], degradation of cytoskeletal proteins [13], and alteration of expression and/or activity of intracellular molecules which transduce signals for cell growth, survival and proliferation [14–16]. Although the inhibitory effect of mistletoe on cancer cell growth keeps growing, the underlying mechanisms to explain its anti-proliferative activity are not fully studied.

In this study, we aimed to investigate anti-proliferative activity of *Taxillus yadoriki* as one of the mistletoes native in Korea against various cancer cell lines, and to elucidate the potential mechanism associated with its anti-proliferative activity.

## Methods

### Reagents

Dulbecco’s Modified Eagle medium (DMEM)/F-12 1:1 Modified medium (DMEM/F-12) for the cell culture was purchased from Lonza (Walkersville, MD, USA). LiCl, MG132, PD98059, SB230580, SP600125, LY294002, BAY 11–7280, leptomycin B (LMB) and 3-(4,5-dimethylthizaol-2-yl)-2,5-diphenyl tetrazolium bromide (MTT) and N-acetyl-L-cysteine (NAC) were purchased from Sigma Aldrich (St. Louis, MO, USA). Antibodies against cyclin D1, phospho-cyclin D1 (Thr286), HA-tag, CRM1 and β-actin were purchased from Cell Signaling (Bervely, MA, USA). All chemicals were purchased from Fisher Scientific, unless otherwise specified.

### Sample preparation

*Taxillus yadoriki* (TY) parasitic to *Cryptomeria japonica* (CJ), *Neolitsea sericea* (NS), *Prunus serrulata* (PS), *Cinnamomum camphora* (CC) and *Quercus acutissima* (QA), respectively, was collected from Jeju island, Korea and formally identified by Ho Jun Son as a researcher of Forest Medicinal Resources Research Center, Korea. Twenty gram of the branches (B) or leaves (L) from TY-CJ, TY-NS, TY-PS, TY-CC and TY-QA was extracted with 400 ml of 70% ethanol with shaking for 72 h. After 72 h, the ethanol-soluble fraction was filtered and concentrated to approximately 120 ml volume using a vacuum evaporator and then freeze-dried. The ethanol extracts was kept in a refrigerator until use.

### Cell culture and treatment

Human colorectal cancer cell lines (HCT116 and SW480), human breast cancer cell line (MDA-MB-231), human pancreatic cancer cell line (AsPC-1), human non-small cell lung cancer cell line (A549) and human prostate cancer cell line (PC-3) were purchased from Korean Cell Line Bank (Seoul, Korea) and grown in DMEM/F-12 supplemented with 10% fatal bovine serum (FBS), 100 U/ml penicillin and 100 μg/ml streptomycin. The cells were maintained at 37 °C under a humidified atmosphere of 5% CO_2_. The test samples were dissolved in dimethyl sulfoxide (DMSO) and treated to cells. DMSO was used as a vehicle and the final DMSO concentration did not exceed 0.1% (*v*/v).

### Cell proliferation assay

Cell proliferation was evaluated by MTT assay. Briefly, cells were plated at a density of 3 × 10^4^ cells/well in 96-well plate and incubated for 24 h. The cells were treated with the test sample at the indicated concentrations for 24 h. Then, the cells were incubated with 50 μl of MTT solution (1 mg/ml) for an additional 2 h. The resulting crystals were dissolved in DMSO. The formation of formazan was measured by reading absorbance at a wavelength of 570 nm using U*V*/Visible spectrophotometer (Human Cop., Xma-3000PC, Seoul, Korea).

### Cell cycle analysis

HCT116 cells were plated at a density of 1 × 10^6^ cells/well in 6-well plate and incubated for 24 h. The cells were treated with TY-NS-B for 24 h. After then, the cells were dissociated with trypsin, washed in cold PBS and fixed with 70% cold ethanol on ice for 30 min. The suspensions were centrifuged at 1500 rpm for 5 min. The pellets were resuspended in a solution containing 50 μg/ml propidium iodide, 1 mg/ml sodium citrate, 0.3 ml nonidet P-40 and 5 μg/ml RNase A and stayed on ice atleast 40 min. Then the pellets were analyzed by a flow cytometer.

### Isolation of cytosol and nucleus fraction

Cytosol and nuclear fractions of cells were prepared using a nuclear extract kit (Active Motif, Carlsbad, CA, USA) according to the manufacturer’s protocols. Briefly, the cells after treatment were harvested with 1 × cold hypotonic buffer and incubated at 4 °C for 15 min. After adding detergent and vortexing for 10 s, the cells were centrifuged at 14,000 g for 1 min at 4 °C and the supernatants (cytoplasmic fraction) were collected and stored at − 80 °C for further analysis. The cell pellets were used for nuclear fraction collection. Cell pellets were re-suspended with complete lysis buffer by pipetting up and down, and incubated at 4 °C for 30 min under shaking. After 30 min, nuclear suspensions were centrifuged at 14,000 g for 10 min at 4 °C, and the supernatants (nuclear fraction) were stored at − 80 °C for further analysis.

### SDS-PAGE and western blot

Cells were plated at a density of 2 × 10^6^ cells/well in 6-well plate and grown to 80% confluence. After treatment, the cells were washed with 1 × phosphate-buffered saline (PBS), and lysed in radioimmunoprecipitation assay (RIPA) buffer (Boston Bio Products, Ashland, MA, USA) supplemented with protease inhibitor cocktail (Sigma-Aldrich) and phosphatase inhibitor cocktail (Sigma-Aldrich), and centrifuged at 15,000 × rpm for 10 min at 4 °C. Protein concentration was determined by the bicinchoninic acid (BCA) protein assay (Pierce, Rockford, IL, USA). The proteins were separated on SDS-PAGE and transferred to PVDF membrane (Bio-Rad Laboratories, Inc., Hercules, CA, USA). The membranes were blocked for non-specific binding with 5% non-fat dry milk in Tris-buffered saline containing 0.05% Tween 20 (TBS-T) for 1 h at room temperature and then incubated with specific primary antibodies in 5% non-fat dry milk at 4 °C overnight. After three washes with TBS-T, the blots were incubated with horse radish peroxidase (HRP)-conjugated immunoglobulin G (IgG) for 1 h at room temperature and chemiluminescence was detected with ECL Western blotting substrate (Amersham Biosciences, Piscataway, NJ, USA) and visualized in Polaroid film.

### Reverse transcriptase-polymerase chain reaction (RT-PCR)

After treatment, total RNA was prepared using a RNeasy Mini Kit (Qiagen, Valencia, CA, USA) and total RNA (1 μg) was reverse-transcribed using a Verso cDNA Kit (Thermo Scientific, Pittsburgh, PA, USA) according to the manufacturer’s protocol for cDNA synthesis. PCR was carried out using PCR Master Mix Kit (Promega, Madison, WI, USA) with human primers for cyclin D1 and GAPDH as followed: cyclin D1: forward 5′-aactacctggaccgcttcct-3′ and reverse 5′-ccacttgagcttgttcacca-3′, GAPDH: forward 5′-acccagaagactgtggatgg-3′ and reverse 5′-ttctagacggcaggtcaggt-3′. The following PCR reaction conditions were used: 1 cycle of (3 min at 94 °C for denaturation), 25 cycles of (30 s at 94 °C for denaturation, 30 s at 60 °C for annealing, and 30 s at 72 °C for elongation), and 1 cycle of (5 min for extension at 72 °C).

### Expression vectors

HA-tagged wild type cyclin D1 and HA-tagged T286A cyclin D1 were provided from Addgene (Cambridge, MA, USA). Transient transfection of the vectors was performed using the PolyJet DNA transfection reagent (SignaGen Laboratories, Ijamsville, MD, USA) according to the manufacturers’ instruction.

### Statistical analysis

All the data are shown as mean ± SEM (standard error of mean). Statistical analysis was performed with one-way ANOVA followed by Dunnett’s test. Differences with *P < 0.05 were considered statistically significant.

## Results

### Comparison of the inhibition of the cell growth by the ethanol extracts from *Taxillus yadoriki* according to the host tree species and plant parts

**Fig. 1:**
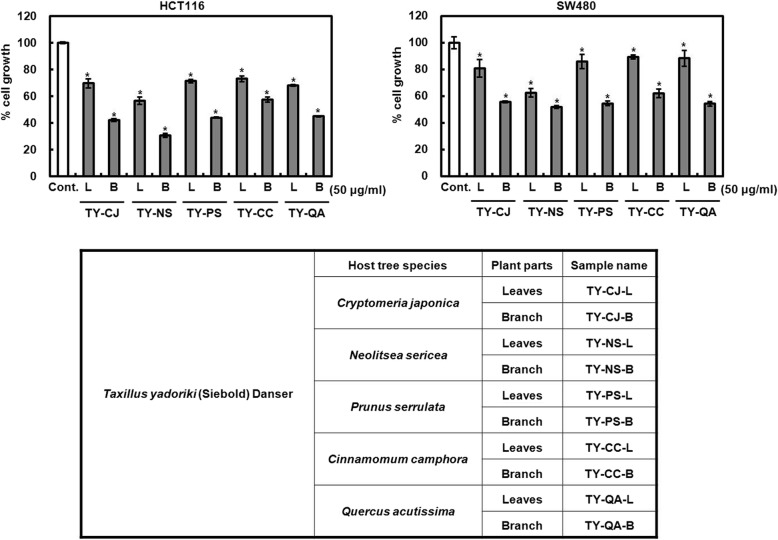
Comparison of anti-proliferative effect of TY from the host trees and plant parts. HCT116 and SW480 cells were plated overnight and then treated with the samples for 24 h. Cell growth was measured using MTT assay. *P < 0.05 compared to cell without the sample treatment

Since *Taxillus yadoriki* (TY) as one of the mistletoes is parasitic to various host trees, we hypothesized that TY’s anti-cancer activity may be different according to the host tree species. Thus, we performed the comparative study of TY on anti-proliferation according to the host tree species. As shown in Fig. 1, the anti-proliferative effect of TY parasitic to *Neolitsea sericea* (NS) was highest in the host tree species such as *Cryptomeria japonica* (CJ), *Neolitsea sericea* (NS), *Prunus serrulata* (PS), *Cinnamomum camphora* (CC) and *Quercus acutissima* (QA). In addition, we observed that the branch (B) of TY is higher than leaves (L) in anti-proliferative effect. Thus, we selected the branch of *Taxillus yadoriki* parasitic to *Neolitsea sericea* (TY-NS-B) for the further study.

### Effect of TY-NS-B on the proliferation of cancer cells and cyclin D1 expression

**Fig. 2:**
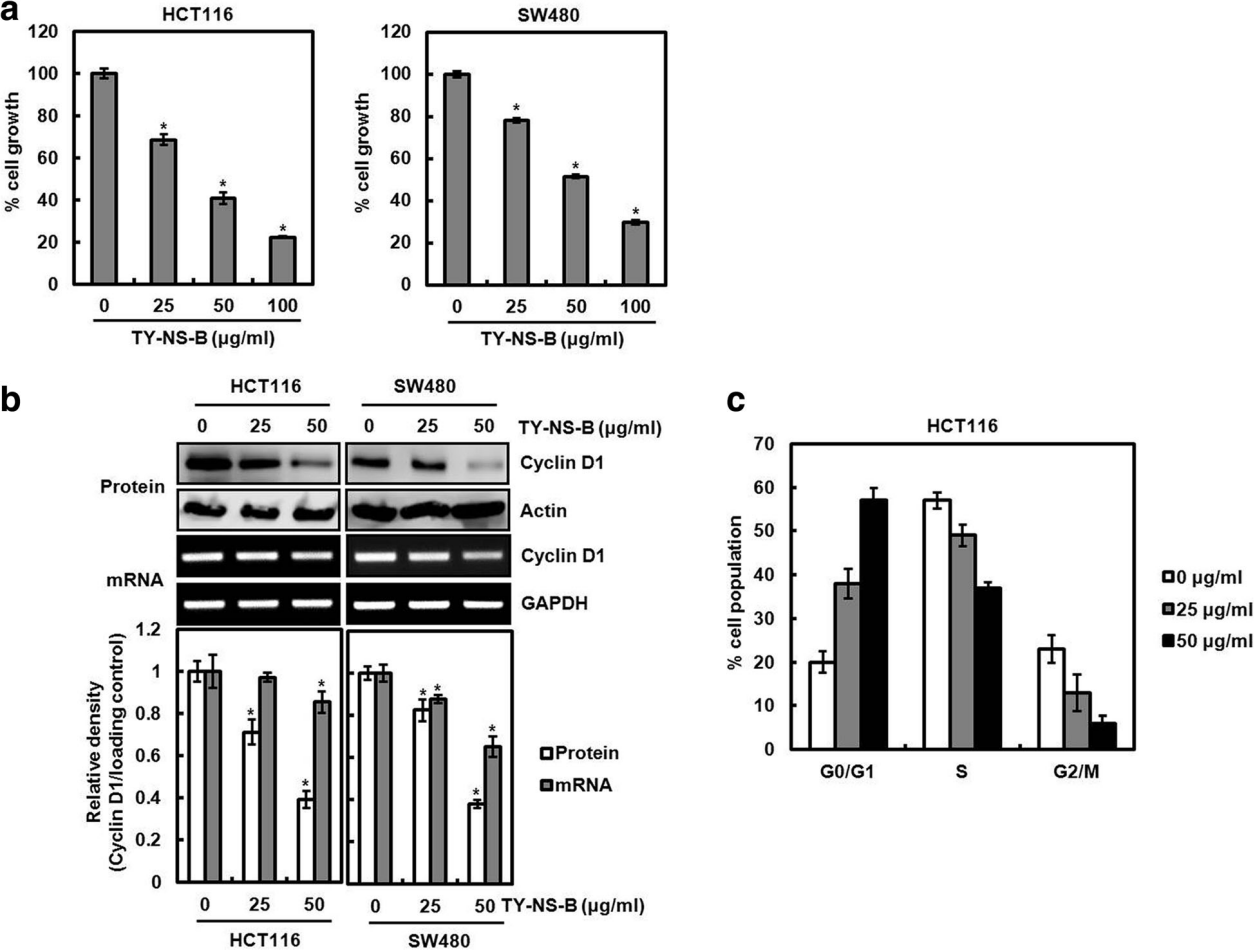
The effect of TY-NS-B on the cell growth and cyclin D1 expression in human colorectal cancer cells. **a** HCT116 and SW480 cells were plated overnight and then treated with TY-NS-B at the indicated concentrations for 24 h. Cell growth was measured using MTT assay. Data represent mean ± SD for three independent experiments. *P < 0.05 compared to cell without TY-NS-B. **b** HCT116 and SW480 cells were plated overnight and then treated with TY-NS-B at the indicated concentrations for 24 h. For Western blot analysis, cell lysates were subjected to SDS-PAGE and the Western blot was performed using antibody against cyclin D1. Actin was used as internal control for Western blot analysis. For RT-PCR analysis of the gene expression of cyclin D1, total RNA was prepared. GAPDH was used as internal control for RP-PCR. Relative density for Western blot and RT-PCR was measured using the software Un-SCAN-IT gel Version 5.1 (Silk Scientific, Inc., Orem, UT, USA). Data represent mean ± SD for three independent experiments. *P < 0.05 compared to cell without TY-NS-B. **c** HCT116 cells were plated overnight and then treated with TY-NS-B at the indicated concentrations for 24 h. Cell cycle progression was analyzed by flow cytometer

**Fig. 3:**
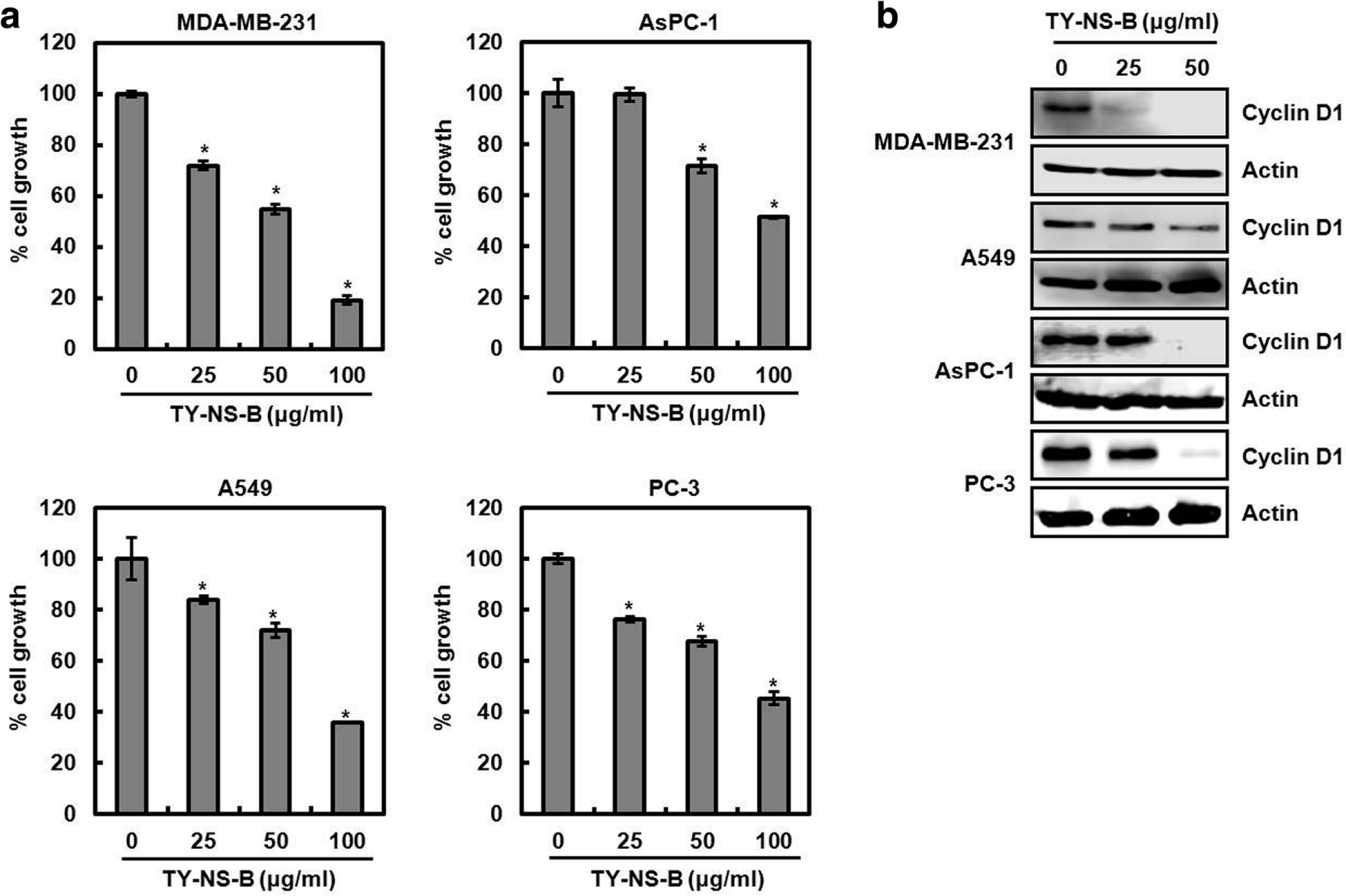
The effect of TY-NS-B on the cell growth and cyclin D1 expression in other cancer cells. **a** Human breast cancer cells (MDA-MB-231), human pancreatic cancer cells (AsPC-1), human non-small cell lung cancer cells (A549) and human prostate cancer cells (PC-3) were plated overnight and then treated with TY-NS-B at the indicated concentrations for 24 h. Cell growth was measured using MTT assay. Data represent mean ± SD for three independent experiments. *P < 0.05 compared to cell without TY-NS-B. **b** MDA-MB-231, AsPC-1, A549 and PC-3 were plated overnight and then treated with TY-NS-B at the indicated concentrations for 24 h. Cell lysates were subjected to SDS-PAGE and the Western blot was performed using antibody against cyclin D1. Actin was used as internal control for Western blot analysis

To investigate whether TY-NS-B affects cancer cells growth, human colorectal cancer cell lines, HCT116 and SW480, were treated with TY-NS-B at the indicated concentrations for 24 h. As shown in Fig. 2a, TY-NS-B dose-dependently induced the cell growth arrest. Because cyclin D1 protein has been regarded as one of the factor associated with the regulation of the cell growth [17], we evaluated that TN-NS-B downregulates cyclin D1 expression. As a result, TN-NS-S decreased cyclin D1 expression in both mRNA and protein level (Fig. 2b). Because cyclin D1 regulates G1/S transition in the cell cycle, we investigated whether the downregulation of cyclin D1 by TY-NS-B contributes to the accumulation of cells in G1 phase. As shown in Fig. 2c, the majority of HCT116 cells without TY-NS-B treatment were in S phase. However, TY-NS-B dose-dependently induced the accumulation of G0/G1 phase in HCT116 cells. In addition, we tested whether TY-NS-B suppresses cell proliferation and cyclin D1 expression in other cancer cells such as MDA-MB-231 (human breast cancer cell line), AsPC-1 (human pancreatic cancer cell line), A549 (human non-small cell lung cancer cell line) and PC-3 (human prostate cancer cell line). As shown in Fig. 3a, TY-NS-B dose-dependently inhibited the growth of MDA-MB-231, AsPC-1, A549 and PC-3 cells. Furthermore, we observed that cyclin D1 protein level was decreased by TY-NS-B treatment in MDA-MB-231, AsPC-1, A549 and PC-3 cells (Fig. 3b).

### TY-NS-B induces cyclin D1 proteasomal degradation

**Fig. 4:**
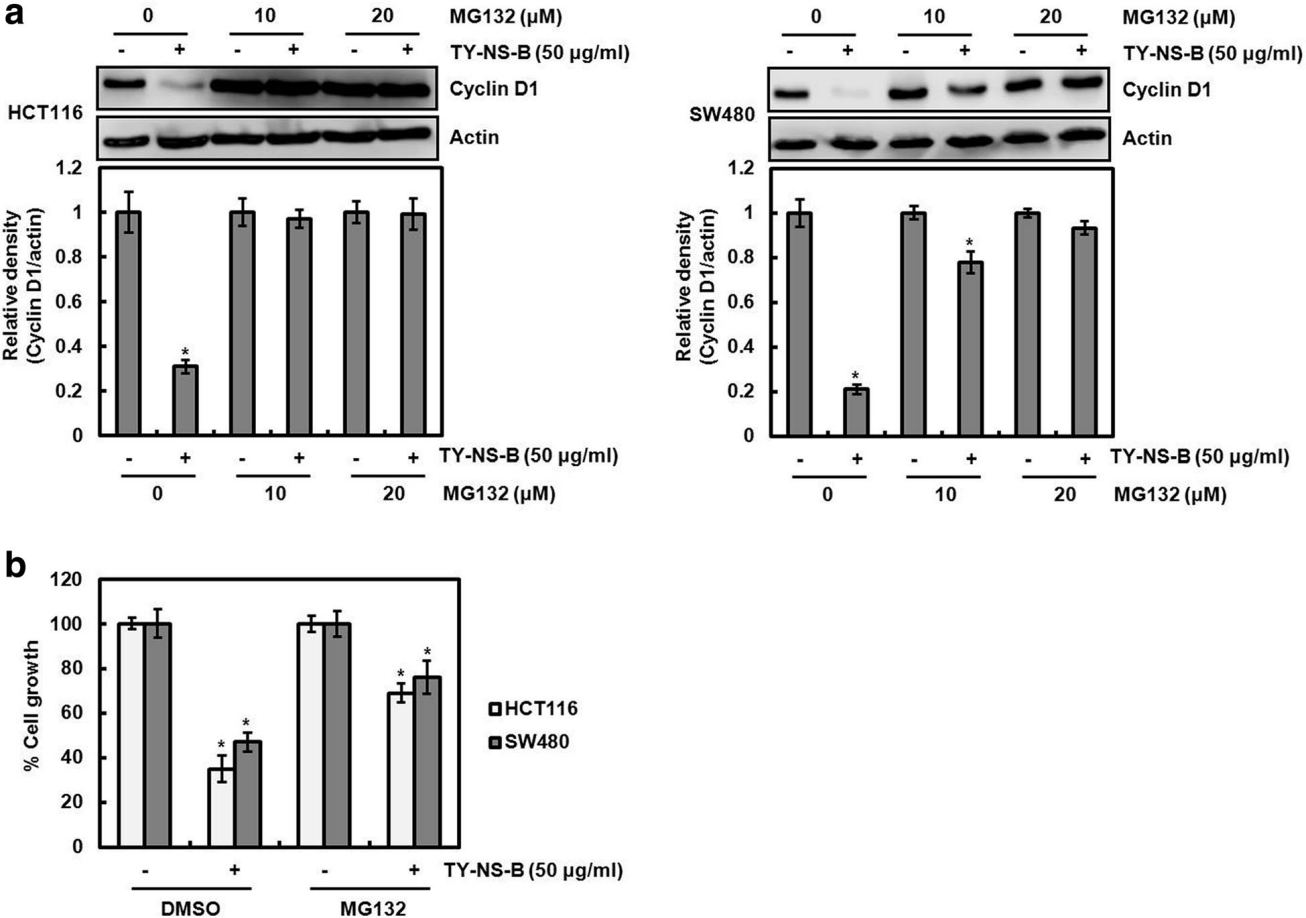
The effect of TY-NS-B on cyclin D1 degradation. **a** HCT116 and SW480 cells were pretreated with MG132 (10 and 20 μM) and then co-treated with TY-NS-B (50 μg/ml). Cell lysates were subjected to SDS-PAGE and the Western blot was performed using antibody against cyclin D1. Actin was used as internal control for Western blot analysis. Relative density for Western blot was measured using the software Un-SCAN-IT gel Version 5.1 (Silk Scientific, Inc). Data represent mean ± SD for three independent experiments. *P < 0.05 compared to cell without TY-NS-B. **b** HCT116 and SW480 cells were pretreated with MG132 (20 μM) and then co-treated with TY-NS-B (50 μg/ml) for 24 h. Cell growth was measured using MTT assay. Data represent mean ± SD for three independent experiments. *P < 0.05 compared to cell without TY-NS-B

We observed that the level change of cyclin D1 protein by TY-NS-B is more dramatic than that of cyclin D1 mRNA (Fig. 2a), which indicates that TY-NS-B may affect cyclin D1 protein stability. To elucidate that the induction of cyclin D1 proteasomal degradation by TY-NS-B, HCT116 and SW480 cells were pretreated with MG132 and then co-treated with TY-NS-B. As shown in Fig. 4, the treatment of TY-NS-B alone downregulated cyclin D1 protein level. However, the presence of MG132 blocked cyclin D1 downregulation mediated by TY-NS-B. In addition, we investigated whether the inhibition of cyclin D1 proteasomal degradation by MG132 affects anti-proliferative effect of TY-NS-B. As shown in Fig. 4b, MG132 treatment attenuated TY-NS-B-mediated inhibition of cell proliferation in HCT116 and SW480 cells. These results indicate that cyclin D1 proteasomal degradation by TY-NS-B may contribute to cyclin D1 downregulation, and subsequently inhibit the cell proliferation.

### Cyclin D1 proteasomal degradation by TY-NS-B is dependent on threonine-286 phosphorylation of cyclin D1

**Fig. 5:**
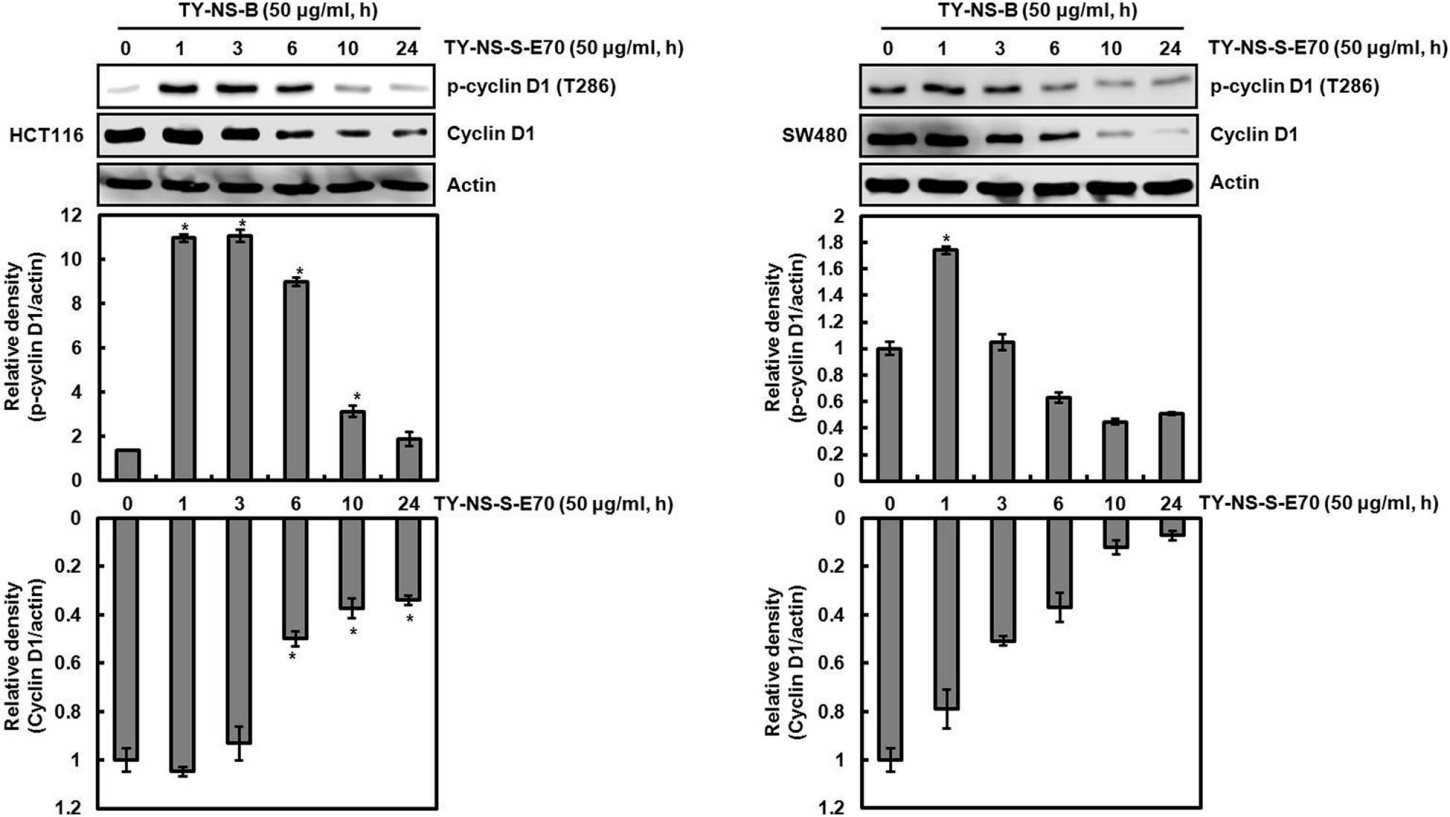
Phosphorylation of T286 of cyclin D1 by TY-NS-B. HCT116 and SW480 cells were plated overnight and then treated with TY-NS-B (50 μg/ml) at the indicated times. Cell lysates were subjected to SDS-PAGE and the Western blot was performed using antibody against p-cyclin D1 (T286) and cyclin D1. Actin was used as internal control for Western blot analysis. Relative density for Western blot was measured using the software Un-SCAN-IT gel Version 5.1 (Silk Scientific, Inc). Data represent mean ± SD for three independent experiments. *P < 0.05 compared to cell without TY-NS-B

**Fig. 6:**
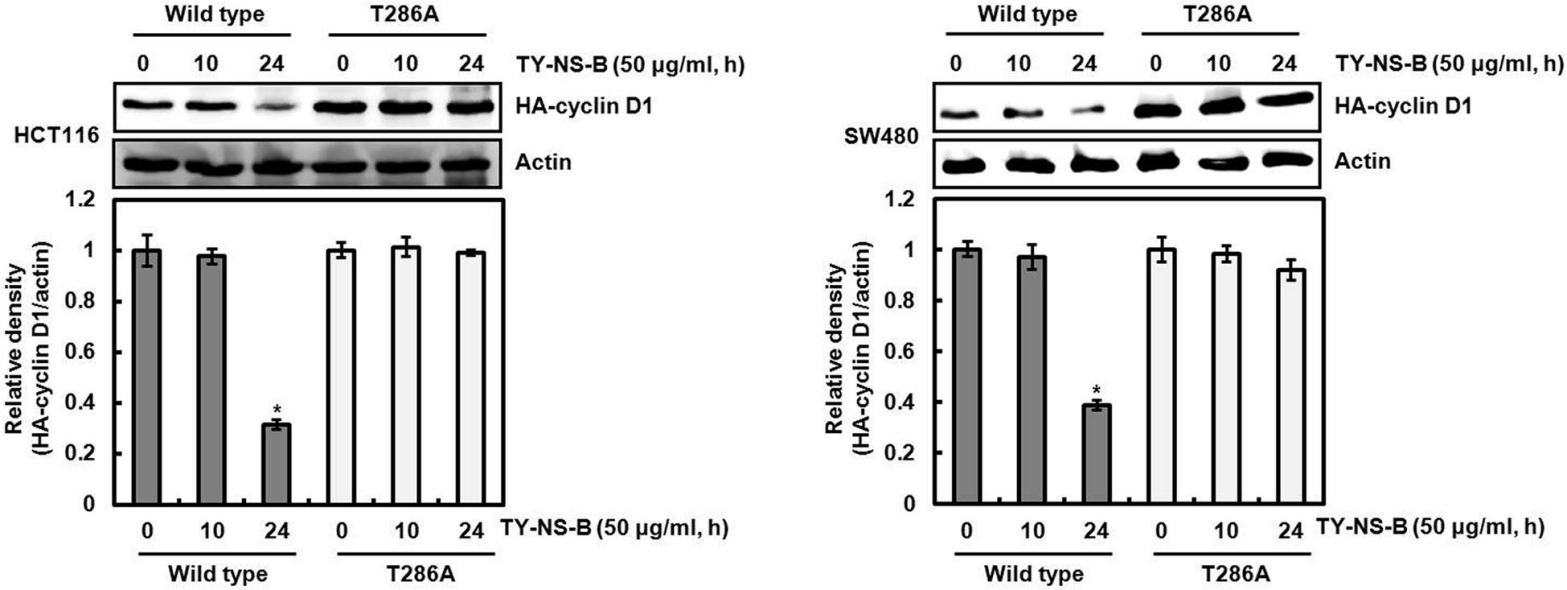
Cyclin D1 degradation by TY-NS-B is dependent on T286 phosphorylation. HCT116 and SW480 cells were transfected with HA-tagged wild type-cyclin D1 or T286A-cyclin D1, and then co-treated with TY-NS-B (50 μg/ml) for the indicated times. Cell lysates were subjected to SDS-PAGE and the Western blot was performed using antibody against HA-cyclin D1. Actin was used as internal control for Western blot analysis. Relative density for Western blot was measured using the software Un-SCAN-IT gel Version 5.1 (Silk Scientific, Inc). Data represent mean ± SD for three independent experiments. *P < 0.05 compared to cell without TY-NS-B

Threonine-286 (T286) phosphorylation of cyclin D1 has been known to be involved in cyclin D1 proteasomal degradation [18]. Thus, we investigated whether TY-NS-B mediates T286 phosphorylation of cyclin D1. As shown in Fig. 5, T286 phosphorylation of cyclin D1 was increased at 1 h after TY-NS-B treatment in both HCT116 and SW480 cells. However, cyclin D1 protein level was significantly decreased at 6 h and 3 h after TY-NS-B treatment in HCT116 and SW480, respectively. These results indicate that T286 phosphorylation of cyclin D1 by TY-NS-B may contribute to cyclin D1 degradation. To confirm whether T286 phosphorylation of cyclin D1 by TY-NS-B is essential to the induction of cyclin D1 proteasomal degradation, HCT116 and SW480 cells were transfected with HA-tagged wild type-cyclin D1 or T286A-cyclin D1, and then treated with TY-NS-B. In this experiment, we observed that TY-NS-B decreases HA-cyclin D1 in the cells transfected with wild type-cyclin D1 at 24 h after treatment, while did not affect the change of HA-cyclin D1 level in the cells transfected with T286A-cyclin D1 (Fig. 6). These findings indicate that cyclin D1 proteasomal degradation by TY-NS-B is dependent on threonine-286 phosphorylation of cyclin D1.

### Determination of the upstream kinases involved in cyclin D1 proteasomal degradation by TY-NS-B

**Fig. 7:**
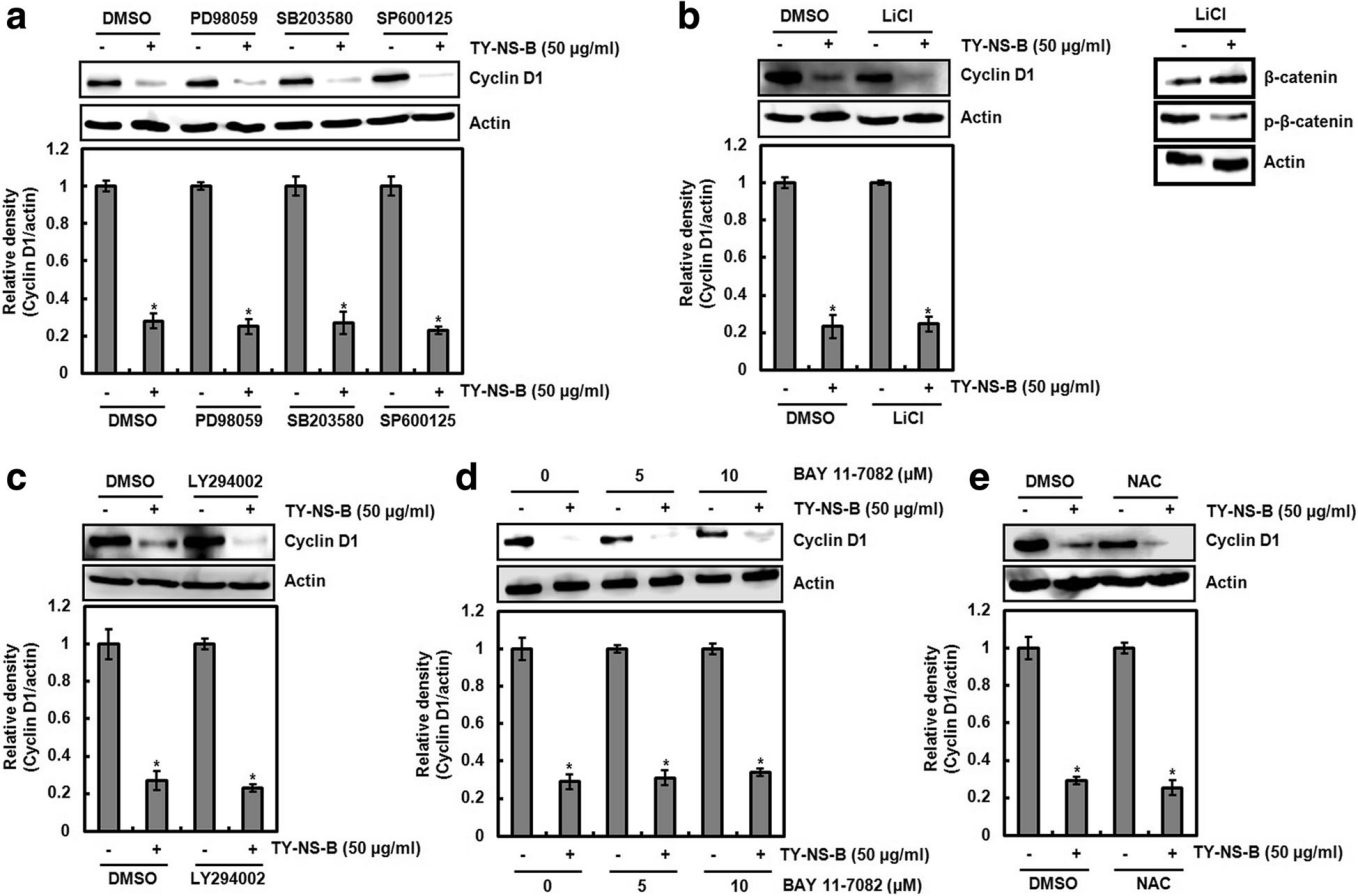
Determination of factors involved in cyclin D1 degradation by TY-NS-B. **a** HCT116 cells were pretreated with 20 μM of PD98059, SB203580 or SP600125. **b** Right panel: HCT116 cells were pretreated with 20 mM of LiCl. Left panel: HCT116 cells were treated with 20 mM of LiCl (**c**) HCT116 cells were pretreated with 20 μM of LY294002. **d** HCT116 cells were pretreated with 5 and10 μM of BAY 11–7082. **e** HCT116 cells were pretreated with 10 mM of NAC. After pretreatment of each inhibitor, HCT116 cells were co-treated with TY-NS-B (50 μg/ml). Cell lysates were subjected to SDS-PAGE and the Western blot was performed using antibody against cyclin D1. Actin was used as internal control for Western blot analysis. Relative density for Western blot was measured using the software Un-SCAN-IT gel Version 5.1 (Silk Scientific, Inc). Data represent mean ± SD for three independent experiments. *P < 0.05 compared to cell without TY-NS-B

T286 phosphorylation and subsequent proteasomal degradation of cyclin D1 has been reported to be regulated by a variety of kinases. To determine the upstream kinases involved in cyclin D1 proteasomal degradation by TY-NS-B, the specific inhibitors such as PD98059 (ERK1/2 inhibitor), SB203580 (p38 inhibitor), SP600125 (JNK inhibitor), LiCl (GSK3β inhibitor), LY294002 (PI3K inhibitor) and BAY 11–7082 (IκK inhibitor) were used. In addition, NAC (ROS scavenger) was used because reactive oxygen species (ROS) has been known to be associated with cyclin D1 degradation [19]. In this study, we observed that TY-NS-B decreases cyclin D1 protein level in both absence and presence of each inhibitor (Fig. 7a-e). Among the upstream kinases involved in cyclin D1 degradation, GSK-3β is reported to be required for cyclin D1 degradation to induce cell cycle arrest [19]. Thus, we evaluated that LiCl as a GSK-3β inhibitor affects the protein and phosphorylation level of β-catenin. As shown in Fig. 7b (left panel), LiCl increased β-catenin protein level by attenuating β-catenin phosphorylation. These results indicate that cyclin D1 proteasomal degradation by TY-NS-B is independent on ERK1/2, p38, JNK, GSK3β, PI3K, IκK and ROS.

### Contribution of cyclin D1 nuclear export by TY-NS-B to cyclin D1 proteasomal degradation

**Fig. 8:**
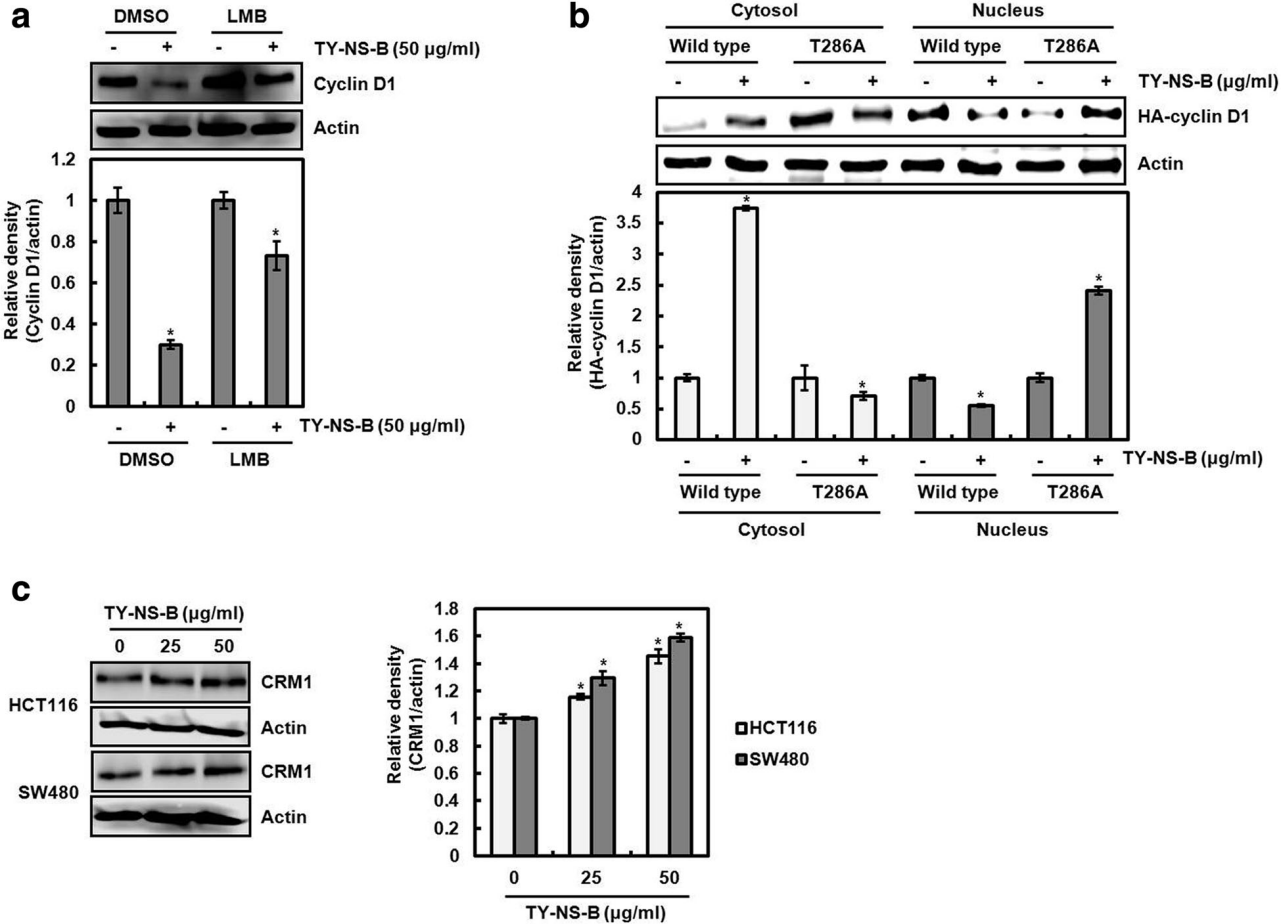
Cyclin D1 nuclear export contributes to cyclin D1 degradation by TY-NS-B. **a** HCT116 cells were pretreated with 50 nM of LMB and then co-treated with TY-NS-B (50 μg/ml). **b** HCT116 cells were transfected with HA-tagged wild type-cyclin D1 or T286A-cyclin D1, and then co-treated with TY-NS-B (50 μg/ml). After treatment, cytoplasm and nucleus were prepared. **c** HCT116 and SW480 cells were treated with TY-NS-B. Cell lysates were subjected to SDS-PAGE and the Western blot was performed using antibody against cyclin D1, HA-cyclin D1 and CRM1. Actin was used as internal control for Western blot analysis. Relative density for Western blot was measured using the software Un-SCAN-IT gel Version 5.1 (Silk Scientific, Inc). Data represent mean ± SD for three independent experiments. *P < 0.05 compared to cell without TY-NS-B

Although we failed to determine the upstream kinases involved in cyclin D1 proteasomal degradation by TY-NS-B, we observed that the treatment of LMB as a nuclear export inhibitor attenuated the downregulation of cyclin D1 by TY-NS-B (Fig. 8a). Indeed, there is growing evidence that T286 phosphorylation of cyclin D1 results in cyclin D1 nuclear export to cytoplasm, which is associated with cyclin D1 proteasomal degradation [20]. Thus, we investigated whether T286 phosphorylation of cyclin D1 by TY-NS-B affects cyclin D1 nuclear export to cytoplasm. As shown in Fig. 8b, cyclin D1 level of the cells transfected with wild type-cyclin D1 was increased in the cytoplasm and decreased in the nucleus by TY-NS-B. However, the transfection of T286A-cyclin D1 blocked cyclin D1 nuclear export by TY-NS-B, resulting in the nuclear accumulation of cyclin D1 level. In addition, we investigated whether TY-NS-B affects the expression of CRM1 as a nuclear exportin. As a result, TY-NS-B dose-dependently increased CRM1 expression. These findings indicate that the increased CRM1 protein by TY-NS-B may export the phosphorylated cyclin D1 at T286 from the nucleus to cytoplasm, which results in cyclin D1 degradation.

## Discussion

Although mistletoe has been used for the cancer therapy, the underlying mechanisms to explain its anticancer activity of the mistletoe are not fully studied. In this study, we firstly compared the anti-proliferative effect of *Taxillus yadoriki* (TY) as one of the mistletoes from the host trees and plant parts. We observed that TY parasitic to *Neolitsea sericea* (NS) is better effective in anti-proliferative effect than that parasitic to *Cryptomeria japonica* (CJ), *Prunus serrulata* (PS), *Cinnamomum camphora* (CC) and *Quercus acutissima* (QA). These data indicates that the difference of anti-proliferative activity of TY may be considered to be due to the kinds of host trees. Indeed, there is growing evidence that the host trees may be an import factor for its bioactivity [21–23]. In addition, we observed that the branch (B) of TY is higher than leaves (L) in anti-proliferative effect. Thus, we selected the branch of *Taxillus yadoriki* parasitic to *Neolitsea sericea* (TY-NS-B) for the further study.

Although cyclin D1 has been viewed as an important regulator of the G1 to S phase transition in normal cells, cyclin D1 also function as a proto-oncogene [24]. Aberrant cyclin D1 overexpression has been regarded to be associated with tumorigenesis and is observed in many different cancer types such as lymphoid, breast, esophageal, lung, colorectal, prostate, pancreas and bladder tumors [24]. Thus, the regulation of cyclin D1 expression has been thought for the potential molecular target of the cancer treatment. In this study, it was observed that cyclin D1 was downregulated by TY-NS-B treatment at both protein and mRNA level in human colorectal cancer cell lines such as HCT116 and SW480. In addition, the cyclin D1 downregulation by TY-NS-B was observed in human breast cancer cells (MDA-MB-231), human pancreatic cancer cells (AsPC-1), human non-small cell lung cancer cells (A549) and human prostate cancer cells (PC-3).

Interestingly, we found that the reduction rate of cyclin D1 protein level by TY-NS-B is more significant than that of cyclin D1 mRNA level. These data indicate that cyclin D1 downregulation of TY-NS-B may be mainly affected by the protein stability although transcriptional inhibition by TY-NS-B of cyclin D1 contributes to cyclin D1 downregulation.

There is growing evidence to support that the increase of cyclin D1 protein stability is responsible for the overexpression of cyclin D1 protein in various human cancers although the amplification of the cyclin D1 gene can account for some, but not all, cases of tumor-specific cyclin D1 overexpression [25]. Indeed, proteasomal degradation has been viewed as an important regulator of cyclin D1 levels in cancer cells [26] and many cancer therapeutic agents exerts anti-proliferative activity through cyclin D1 proteasomal degradation [27–30]. These studies indicate that the induction of cyclin D1 degradation may provide a useful avenue for cancer treatment. Thus, we investigated the effect of TY-NS-B on cyclin D1 proteasomal degradation and observed that MG132 blocks the downregulation of cyclin D1 protein level by TN-NS-B. Because MG132 as a specific proteasome inhibitor has been widely used for the effect of many anticancer agents on the induction of cyclin D1 proteasomal degradation, our data indicates that TY-NS-B may induce cyclin D1 proteasomal degradation.

Threonine-286 phosphorylation (T286) of cyclin D1 has been reported to be associated with its proteasomal degradation and the inhibition of T286 phosphorylation attenuates cyclin D1 proteasomal degradation through the ubiquitin-proteasome pathway [18]. T286 phosphorylation involved in cyclin D1 proteasomal degradation can be regulated by a variety of the kinases such as p38, ERK1/2, JNK, GSK3β, IκK and PI3K [31–35]. In addition, reactive oxygen species (ROS) induces cyclin D1 degradation [19]. In this study, TY-NS-B increased the phosphorylation status of cyclin D1 at T286, and the mutation of threonine-286 to alanine (T286A) attenuated cyclin D1 proteasomal degradation by TY-NS-B. These data indicate that T286 phosphorylation of cyclin D1 may be an essential step for cyclin D1 proteasomal degradation by TY-NS-B. However, we failed to determine the factor involved in the induction of cyclin D1 proteasomal degradation by TY-NS-B. Our data showed that the inhibition of p38, ERK1/2, JNK, GSK3β, IκK, PI3K and ROS did not affect the downregulation of cyclin D1 by TY-NS-B, which indicates that the downregulation of cyclin D1 by TY-NS-B may be independent of p38, ERK1/2, JNK, GSK3β, IκK, PI3K and ROS. Thus, the determination of the factor involved in the induction of cyclin D1 proteasomal degradation by TY-NS-B is required in the further study.

Interestingly, we found that LMB as a nuclear export inhibitor suppresses the downregulation of cyclin D1 protein level by TY-NS-B. It has been reported that T286 phosphorylation of cyclin D1 contributes to redistribution of cyclin D1 from the nucleus to the cytoplasm, and T286 phosphorylation-dependent degradation of cyclin D1 is accompanied by its relocation to the cytoplasm [36]. Cytoplasmic cyclin D1 is translocated into the nucleus in association with its binding partners, which increases its oncogenic potential [37]. However, T286 phosphorylation of the nuclear cyclin D1 promotes the association with the nuclear exportin, CRM1, resulting to redistribution of cyclin D1 from the nucleus to the cytoplasm and rapid degradation within the cytoplasm [20]. In this study, we determined that TY-NS-B increases cytoplasmic cyclin D1 protein level and decreases nuclear cyclin D1 protein level in the cells transfected with wild type-cyclin D1 compared to the cells transfected with T286A-cyclin D1. In addition, we observed that TY-NS-B dose-dependently increases CRM1 expression. These data indicates that cyclin D1 export from the nucleus to cytoplasm may contribute to its degradation by TY-NS-B.

## Conclusion

In conclusion, TY-NS-B downregulates the level of cyclin D1 protein through the proteasomal degradation via T286 phosphorylation-dependent cyclin D1 export from nucleus to cytoplasm. This study supports that TY-NS-B suppresses the proliferation of cancer cells, and downregulation of cyclin D1 may play a role in TY-NS-B-induced anti-cancer activity. Our findings will provide the potential TY-NS-B usage in the cancer drug development.

## Abbreviations

CC: *Cinnamomum camphora*
CJ: *Cryptomeria japonica*
DMSO: Dimethyl sulfoxide
ERK1/2: Extracellular signal–regulated kinase 1/2
GSK3β: Glycogen synthase kinase 3β
IκK: IκB kinase
JNK: C-Jun N-terminal kinases
LMB: Leptomycin B
MTT: 3-(4,5-dimethylthizaol-2-yl)-2,5-diphenyl tetrazolium bromide
NAC: N-acetyl-L-cysteine
NS: *Neolitsea sericea*
PI3K: Phosphoinositide 3-kinase
PS: *Prunus serrulata*
QA: *Quercus acutissima*
ROS: Reactive oxygen species
RT-PCR: Reverse transcriptase-polymerase chain reaction
T286: Threonine-286
T286A: Threonine-286 to alanine
TY: *Taxillus yadoriki*
TY-NS-B: Branch of *Taxillus yadoriki* parasitic to *Neolitsea sericea*
